# Natural language processing to classify electrocardiograms in patients with syncope: A preliminary study

**DOI:** 10.1002/hsr2.904

**Published:** 2022-10-31

**Authors:** James Quinn, David Kim, Brian Travis Rice, Wei David Hao

**Affiliations:** ^1^ Department of Emergency Medicine Stanford University California Stanford USA

**Keywords:** classification, electrocardiograms, natural language processing, syncope

## 1 INTRODUCTION

Syncope is a dramatic symptom, accounting for about 1.2% of all ED visits.^1^, ^2^ An electrocardiogram (ECG) is recommended in these patients.^3^, ^4^ Clinical risk stratification of patients with syncope includes interpretation of ECG findings.^1^, ^5^, ^6^, ^7^, ^8^, ^9^ In risk stratification studies an “abnormal” ECG is consistently the most important risk factor, especially when considering the risk of cardiac arrythmia and/or sudden death. The characteristics of an “abnormal” ECG are similar in these studies. Where differences exist, they are around numerical cut points and criteria requiring subjective interpretation. Furthermore, few studies have considered the knowledge, interpretation, and time needed to apply criteria at the bedside.^7^ A consensus conference of experts determined 11 important characteristics of an “abnormal” ECG.^10^, ^11^ All these characteristics are automatically generated and reported by ECG machines.

In this study, we use natural language processing (NLP) to extract the 11 syncope‐specific criteria from a machine‐generated ECG report and use them to classify abnormal ECGs for syncope. We compare this classification to unstructured physician interpretation as well as the general summary classification of the machine report.

## 2 METHODS

Commonly used decision tools and expert guidelines were reviewed to come up with criteria for an “abnormal” syncope ECG. Table 1 summarizes the studies and guidelines. From this, we determined that any of the following 11 criteria as indicative of an “abnormal” ECG in a patient with syncope; QTc > 470, LBBB, QRS > 100, *Q* waves, ST segment changes, PR < 120 ms, any AV (Type I, II, III), left axis deviation, non‐sinus rhythm (including paced) multiple PVC's, sinus bradycardia <40.

**Table 1:** Syncope high‐risk ECG criteria from risk stratification studies

QTc > 470
LBBB
QRS > 100
PR < 120 ms
Any AV (Type I, II, III)
Left axis deviation
Non‐sinus rhythm (including paced) multiple PVC's
Sinus bradycardia < 40
Q waves
ST segment changes

*Note*: Based on decision rules and consensus groups for “high‐risk” ECG abnormalities.^1^, ^5^, ^6^, ^7^, ^8^, ^9^, ^10^, ^11^

Abbreviation: ECG, electrocardiogram.

**Figure 1:**
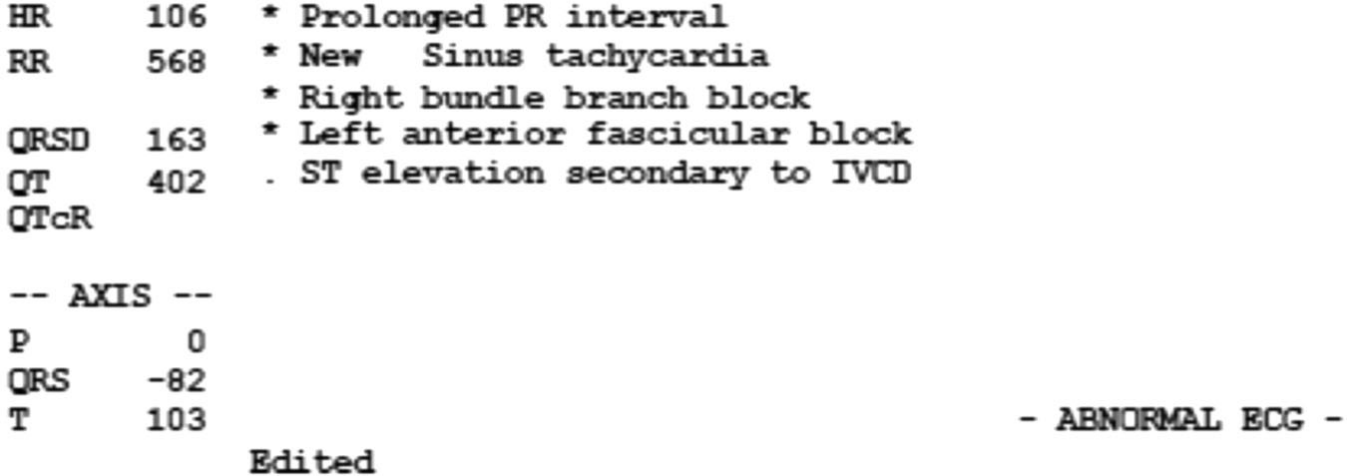
Example of ECG report processed with natural language processing. ECG, electrocardiogram.

An NLP algorithm was written in Python to extract the criteria from a typical ECG report (Figure 1). The report was extracted in XML format from the standard 12 lead ECG. The report was interpreted by the NLP algorithm and classified as abnormal if any “abnormal” characteristic was present.

To test and refine the algorithm it was applied to the first ECG from a random sample of 100 ED visits for syncope. These 100 ECGs also underwent precise manual application of the 1100 criteria. The manual application was considered the gold standard for accuracy and classification. The NLP algorithm was applied after refinement and used to classify ECGs as abnormal based on these criteria. The performance of the final refined NLP algorithm used to classify the ECG was assessed against the machine‐generated summary report and experienced physician interpretation. In the case of the machine‐generated summary report, it was classified as “normal/borderline” versus “other/abnormal.” The ECGs were also evaluated by two experienced board‐certified emergency medicine physicians. The physicians were aware that the ECG came from patients presenting to the emergency department with syncope but given no specific criteria to apply. They were asked to classify the ECGs as “normal” versus “abnormal” in the cases that they felt the ECG had a finding concerning for syncope. Accuracy, sensitivity, and specificity were calculated with 95% confidence intervals. All data were de‐identified by the institutions health information system before being provided to the researchers for analysis. The protocol was approved by researchers a *t* and the protocol was approved by the Stanford University Institutional Review Board with an exemption from informed consent.

## 3 RESULTS

The initial application and assessment of the NLP and script involved 1100 criteria from 100 ECGs of which 62% had at least one “abnormal” criterion. The initial algorithm application correctly classified 1090 of 1100 criteria resulting in 99% (95% CI: 98%–100%) accuracy. The 10 incorrect interpretations across seven ECGs resulted in one incorrectly classified ECG. Rescripting produced a refined algorithm with 100% accuracy and no incorrect categorizations. The refined NLP algorithm had 100% sensitivity (95% CI: 94%–100%) and 100% specificity (95% CI: 91%–100%) compared to the machine‐generated ECG report, 87% sensitivity (95% CI: 76%–94%) and 54% specificity (95% CI: 37%–69%) and physician determinations, sensitivities of 57% (95% CI: 43%–69%) and 85% (95% CI: 74%–93%), specificities of 76% (95% CI: 60%–89%) and 84% (95% CI: 70%–94%) (Table 2).

**Table 2:** Comparison of high‐risk syncope ECG classification

Method	Sensitivity (95% CI)	Specificity (95% CI)
NLP classification	100% (94%–100%)	100% (99%–100%)
ECG report	87% (76%–94%)	54% (37%–69%)
Physician 1	85% (74%–93%)	84% (70%–94%)
Physician 2	57% (43%–69%)	76% (60%–89%)

Abbreviations: ECG, electrocardiogram; NLP, natural language processing.

## 4 DISCUSSION

In this study, we showed that NLP of a standard ECG report can accurately identify 11 predetermined ECG criteria for syncope. After identifying the criteria, they were utilized to correctly classify abnormal ECGs. The process does not require interpretation of the ECG waveforms and is faster and more accurate than experienced emergency physicians. The process may improve the bedside assessment of ECG criteria for syncope and the performance of clinical decision tools.

A standard 12 lead ECG comes with the waveforms and a machine‐generated report based on a computer algorithm used to interpret the raw ECG data. The use of computers to interpret ECG goes back to 1961 with automated reports becoming standard since the late 1970s.^12^, ^13^, ^14^ Over the years, algorithm techniques and more data from more leads have improved the accuracy of the reports.^15^ Current machine‐generated reports are very accurate with small variations between different manufacturers. These differences usually involve interval measurements and interpretation.^16^ Traditionally machine‐generated reports are usually overread by physicians for accuracy and there are guidelines for their use. More recently the machine‐generated reports have been found useful for triage decisions in Emergency Departments.^17^, ^18^

In this study, we performed NLP techniques on the automated report and used manual interpretation of the variables as the gold standard. The initial scripting had 10 incorrect interpretations. Four of these were interval measurement discrepancies. Specifically, the automated report did not note first‐degree AVB as detailed on the ECG with a PR interval greater than 200 ms. These were easily rescripted using the numerical output and not the written report for this criterion. Other misses were due to spelling the entire word “premature ventricular contractions” versus PVC and one miss had both atrial fibrillation and sinus rhythm in the report. The features missed although recurrent were rare and easily rescripted to improve accuracy.

The preliminary development of the NLP algorithm involved the assessment of 1100 manually checked criteria but was limited to 100 ECGs and only one type of machine‐generated report. Further testing and interpretation of other machine‐generated reports are warranted. However, with the standardization of machines and reports, we would expect little or no differences in our findings. For the purposes of NLP scripting, any automated report that could be converted to XML format could be interpreted and classified. If these automated report and format could be imported into a bedside clinical application widespread validation and implementation could be undertaken.

## 5 CONCLUSION

We report the preliminary findings of a simple NLP algorithm that can be applied to an ECG machine automated report to allow near‐perfect classification of abnormal syncope ECGs. This NLP algorithm may be a valuable tool to help accurately interpret ECGs in patients with syncope and improve their risk stratification.

## AUTHOR CONTRIBUTIONS

**James Quinn**: Conceptualization; Formal analysis; Methodology; Supervision; Writing – original draft. **David Kim**: Data curation; Formal analysis; Methodology; Writing – review & editing. **Brian Travis Rice**: Writing – review & editing. **Wei David Hao**: Investigation; Writing – review & editing.

## ETHICS STATEMENT

The Stanford University Institutional Review Board with an exemption from informed consent under the policies of the US Federal Policy for the Protection of Human Subjects Research.

## TRANSPARENCY STATEMENT

The lead author James Quinn affirms that this manuscript is an honest, accurate, and transparent account of the study being reported; that no important aspects of the study have been omitted; and that any discrepancies from the study as planned (and, if relevant, registered) have been explained.

## Data Availability

The data that support the findings of this study are available from the corresponding author upon reasonable request.
